# PD237 Perspectives On Involvement In Health Technology Assessment: Insights From Representatives Of Persons With Mental Health Conditions

**DOI:** 10.1017/S0266462324004549

**Published:** 2025-01-07

**Authors:** Jorge Alonso López, María José Faraldo Vallés, Patricia Gómez-Salgado

## Abstract

**Introduction:**

The participation of people with mental health conditions or patient representatives is of relevance in the health technology assessment (HTA) field, although there are some challenges that need to be considered. This study was designed to gain the perspectives of patient representatives with experience participating in HTA projects, with the aim of delving into the main issues and proposing ideas for the future.

**Methods:**

A structured interview, based on five open-ended questions, was formulated and distributed via email. Five patient representatives from the Saúde Mental FEAFES Galicia (the Federation of Associations of Family Members and Persons with Mental Illness of Galicia, Spain) who had actively engaged in HTA projects over the past five years participated in the study. The collected data were analyzed using thematic analysis.

**Results:**

Participants underscored the pivotal role of involving persons with mental health conditions and their representatives in HTA and emphasized the importance of knowing their perspectives, preferences, and values. Challenges included the complexity of HTA reports and processes, in terms of length and the technical language used, and socioeconomic barriers. Proposed solutions included material adaptation, streamlined processes, and institutional and professional support. Future recommendations emphasized improving awareness, disseminating the HTA field, fostering active participation, and emphasizing the relevance of participating in the HTA process. Of particular significance was the encouragement of training and capacity building among patients with mental health conditions.

**Conclusions:**

This study reveals the need to strengthen and facilitate the participation of individuals with mental health conditions in HTA processes, and emphasizes the importance of knowing their perspectives, preferences, and values. Recommendations include training and capacity building, simplifying materials and processes, and providing adequate support.

